# From Structure to Vulnerability: Mitochondrial Supercomplexes in Cancer Cells

**DOI:** 10.3390/cells15030258

**Published:** 2026-01-29

**Authors:** Corinne E. Griguer, Susanne Flor, Claudia R. Oliva

**Affiliations:** Free Radical & Radiation Biology Program, Department of Radiation Oncology, University of Iowa, Iowa City, IA 52242, USA; susanne-flor@uiowa.edu (S.F.); claudia-oliva@uiowa.edu (C.R.O.)

**Keywords:** mitochondrial supercomplexes, cancer metabolism, respiratory chain assembly factors, therapeutic resistance, bioenergetics

## Abstract

**Highlights:**

**What are the main findings?**

- Mitochondrial respiratory supercomplexes (SCs) are dynamically organized assemblies regulated by membrane lipid composition and specific protein factors.
- SCs formation optimizes electron transfer efficiency and modulates mitochondrial reactive oxygen species production.

**What are the implications of the main findings?**

- Dynamic SCs remodeling contributes to mitochondrial adaptation under metabolic and environmental stress.
- In cancer, SCs dynamics support metabolic flexibility and redox homeostasis and may present therapeutic opportunities.

**Abstract:**

Mitochondrial respiratory supercomplexes are emerging as key regulators of bioenergetics, redox homeostasis, and metabolic plasticity in cancer. Their assembly enhances electron transport efficiency, limits reactive oxygen species production, and supports the high oxidative and biosynthetic demands of tumor growth. Cancer cells remodel supercomplex organization in response to hypoxia, nutrient limitation, and therapeutic stress, enabling rapid metabolic adaptation. Multiple assembly factors—including COX subunits, HIGD1A/2A, COX7A2L (SCAF1), cardiolipin remodeling enzymes, and Complex I assembly factors such as NDUFAF1 and NDUFAF2—contribute to supercomplex stabilization and can be dysregulated in malignancy. Alterations in these factors enhance respiratory flexibility and therapy resistance, particularly in aggressive tumors such as glioblastoma. However, critical gaps remain, including incomplete understanding of the molecular mechanisms controlling supercomplex assembly and remodeling, limited validation of functional findings in primary patient-derived cells or clinical samples, and uncertainty regarding the contribution of supercomplex to therapy resistance and metabolic adaptation across tumor types. Advances in structural biology and functional imaging have uncovered tumor-specific vulnerabilities within supercomplex architecture that may be exploited therapeutically. Targeting supercomplex assembly, cardiolipin–protein interactions, or electron flux through individual supercomplex modules represents a promising approach to disrupt cancer metabolism and sensitize tumors to treatment. This review synthesizes current knowledge on supercomplex regulation, function, and therapeutic potential in cancer, and outlines key unanswered questions that remain to be addressed.

## 1. Introduction

Mitochondria are critical organelles in eukaryotic cells, responsible for generating cellular energy, maintaining metabolic balance, and regulating homeostasis. The central function of mitochondria is to produce adenosine triphosphate (ATP), the primary energy currency of the cell, through a process known as oxidative phosphorylation (OXPHOS). OXPHOS takes place across the inner mitochondrial membrane (IMM), where under normoxic conditions, electrons derived from glycolysis and the Krebs cycle are shuttled through the four complexes of the electron transport chain (ETC) in a series of redox reactions. In mammals, the ETC is largely composed of four protein complexes embedded in the IMM—complex I (CI; NADH–ubiquinone oxidoreductase), complex II (CII; succinate–quinone oxidoreductase), complex III (CIII; ubiquinol–cytochrome c oxidoreductase), and complex IV (CIV; cytochrome c oxidase)–each of which is composed of multiple subunits encoded by nuclear and mitochondrial DNA. Electrons are shuttled into the ETC by NADH, which reduces CI, and by FADH2, which reduces CII. Coenzyme Q then mediates the transfer of electrons from CI and CII to CIII, and cytochrome c transfers the electrons from CIII to CIV [1,2]. Electron transfer from CI, but not from CII, creates a proton gradient and, thus, an electrochemical potential across the IMM, which is ultimately harnessed by ATP synthase (complex V; CV) to synthesize ATP, completing the mitochondrial respiratory chain [3].

While NADH is generated by the glycolytic production of pyruvate, the largest amount of NADH is produced by the redox reactions of the Krebs cycle, in which acetyl CoA is metabolized to oxaloacetate. When sufficient glucose is available, the Krebs cycle is fueled largely by the glycolytic production of pyruvate, which is oxidized to acetyl CoA. However, the Krebs cycle is also fueled by the β-oxidation of fatty acids, which produces acetyl CoA, and the metabolism of glutamine to α-ketoglutarate, a Krebs cycle intermediate [4,5,6,7]. When glucose is scarce or the cellular energy demand is dramatically increased, fatty acid β-oxidation and glutaminolysis may be upregulated to support OXPHOS. FADH_2_ is primarily produced when succinate is oxidized to fumarate in the Krebs cycle, although it is also produced by fatty acid β-oxidation [6,8,9].

In addition to ensuring the bioenergetic demands of cells are met, the ETC contributes to the production of reactive oxygen species (ROS). Alterations that reduce the efficiency of electron transport can increase electron leak from CI and CIII, leading to the elevated production of ROS, which can damage cellular components and activate transcription factors with pathologic ramifications [8,9,10,11]. ROS also regulate the induction of antioxidant enzymes, however, as well as the cellular response to environmental stress, including nutrient deprivation, hypoxia, and therapeutic toxicity, so the process of electron transport through the ETC must be tightly controlled to maintain homeostasis [12]. Such control and, therefore, the metabolic flexibility necessary to ensure cell survival may be mediated by the assembly of individual ETC complexes into higher-order mitochondrial supercomplexes (SCs).

## 2. From Fluid to Plasticity: Evolving Models of Respiratory Chain Organization in Mammalian Mitochondria

Historically, two main models have described the organization of the ETC complexes. The “fluid model” suggests that all ETC complexes are freely mobile in the IMM, and electron transfer occurs through random diffusion-mediated collisions [13]. This fluid model dominated until 2001 when, using mild detergent-based mitochondrial membrane solubilization and blue native PAGE (BN-PAGE), Cruciat et al. [14] and Schägger & Pfeiffer [15] demonstrated the existence of SCs consisting of two or more ETC complexes in varying stoichiometries, supporting a “solid-state model.” The existence of SCs across multiple species—from bacteria to humans—suggests a conserved evolutionary advantage of this organization.

**Figure 1:**
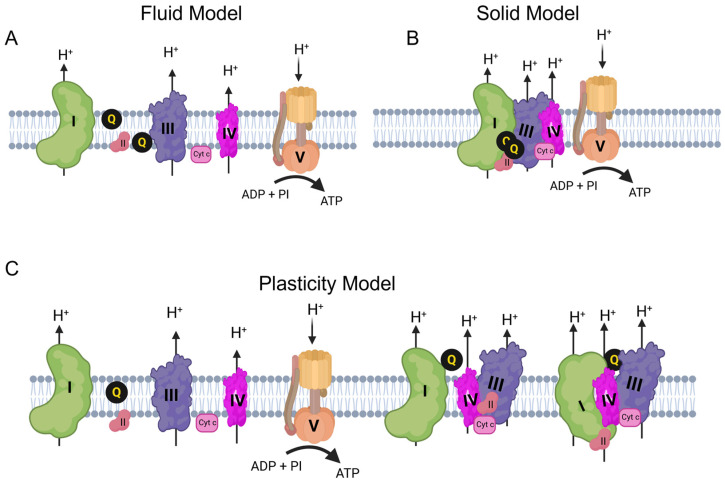
Models of mitochondrial electron transport chain organization. Schematic representation of the three proposed models of respiratory chain organization in the inner mitochondrial membrane. (**A**) Fluid model: Individual complexes diffuse freely and interact transiently. (**B**) Solid model: Complexes are arranged in stable, rigid assemblies that channel substrates efficiently and minimize electron leak. (**C**) Plasticity model: Combines features of both models, with a dynamic equilibrium between free complexes and stable SCs that changes in response to metabolic state, stress, and tissue-specific demands. Together, these models illustrate the structural and functional diversity of ETC organization across physiological and pathological conditions. Created BioRender.com. Created in BioRender. Oliva CR (2025) https://app.biorender.com/illustrations/695ff818fc4c6c07b933ef21 (accessed on 9 January 2026).

A more nuanced view of ETC complex organization, the “plasticity model,” has now been proposed, however [16]. Incorporating elements of both earlier theories, the “plasticity model” suggests that ETC complexes exist in dynamic equilibrium between the free and assembled states (Figure 1). Although direct in vivo evidence of this dynamic interconversion remains somewhat limited, this model is further supported by accumulating evidence that the assembly of SCs provides mitochondria with greater flexibility to respond to metabolic demands, enabling rapid adaptation to change in energy requirements. Indeed, evidence suggests that the plasticity of ETC complex organization is particularly relevant in tissues with high energy demands, and aberrant SCs assembly has been implicated in various diseases in which mitochondrial dysfunction is a hallmark [13,16,17,18]. Conversely, tumor cells and cancer stem cells likely exploit this plasticity to support cell survival, growth, and metastasis in various environmental conditions. This review explores the emerging role of mitochondrial SCs in cancer, highlighting their structural organization and functional significance. We focus on the mechanisms by which SCs support tumor metabolism and contribute to therapy resistance and, therefore, represent potential targets for novel anticancer strategies.

## 3. Structural Organization and Functional Significance of Supercomplexes

The presence and structural organization of mitochondrial SCs vary across and even within species, with evidence strongly suggesting further tailoring according to tissue type and physiologic state [19,20,21]. For instance, heart and skeletal muscle, tissues requiring high, sustained energy, show a prevalence of SCs with configurations optimized for efficient ATP production, whereas liver and other metabolic tissues exhibit different configurations more suited to diverse metabolic roles. In addition, acute cellular stress affects the assembly and configuration of SCs [22,23]. Consistent with this concept, recent in situ cryo–electron microscopy studies have resolved multiple native respiratory SCs architectures directly within intact mammalian mitochondrial membranes, revealing substantial structural heterogeneity and dynamic organization that varies with cellular context [24].

SCs consisting of CI, CIII, and CIV in various combinations have been identified. Consistent among all SCs is the presence of homodimeric CIII (CIII_2_), but SCs can exist independently of CI or CIV, such as CIII_2_ + CIV_1–2_ and CI + CIII_2_. When present in SCs, CI may be in a monomeric or dimeric state, and CIV may be in a monomeric or multimeric (CIV_2–4_) state [25,26,27]. The minimal SCs composition varies across tissues, but CIII_2_ may serve as the central structural core [20], with CI requiring stabilization via interactions with other complexes. Because it contains each of the ETC complexes required for electron transfer to O_2_, the most abundant SCs in eukaryotes, CI + CIII_2_ + CIV, is referred to as the respirasome [15,21,28,29]. Larger respirasome assemblies, such as CI_2_ + CIII_2_ + CIV_2_, have also been observed by cryo-EM in isolated human mitochondria and, more recently, by in situ structural approaches that preserve native membrane organization [24,30]. Despite its likely importance in mammalian cells, the respirasome is not found in the mitochondria of Saccharomyces cerevisiae which, unlike mammals and at least one other yeast strain, *Yarrowia lipolytica*, lack CI [20,31].

CII is typically not detected within SCs by BN-PAGE, and genetic and pharmacologic inhibition of a CII subunit (SDHC) in cultured mammalian cells similarly indicated that CII is not likely a component of the respirasome but may have a regulatory role in respirasome formation [32]. However, emerging studies using cryo-EM and cross-linked proteomics suggest CII might interact weakly with SCs but dissociate during mitochondrial isolation in the presence of detergents [33], and a CII-containing SC (CI + CII + CIII_2_ + CIV_2_), as identified by cryo-EM and cryo-tomography, has been isolated from the ciliate protist *Tetrahymena thermophila* [16].

The mechanism by which SCs are assembled also remains to be confirmed, with evidence to support two different pathways—either through the association of fully assembled complexes or via stepwise incorporation of subunits into a partially assembled CI scaffold [11,20,34]. The relative rarity of free CI detected in mammalian mitochondria supports the hypothesis that SC assembly stabilizes and supports full CI formation, at least. Structural studies have highlighted particularly stable interactions between CI and CIII_2_ and between CIII_2_ and CIV [22,23].

**Figure 2:**
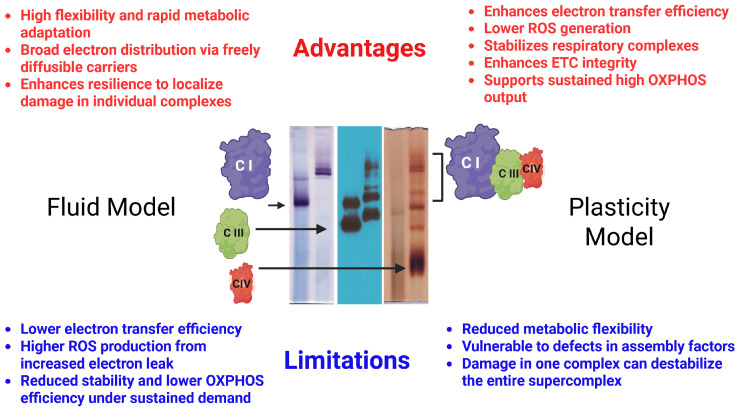
Advantages and limitations of fluid vs. plasticity models of the mitochondrial respiratory chain. BN-PAGE shows individual respiratory complexes (I–IV) and SCs. In the plasticity model, stable SCs promote substrate channeling, efficient electron transfer, and reduced ROS, but may underestimate dynamic flexibility. The fluid model allows freely diffusing complexes and adaptable interactions, but electron transfer efficiency may be lower. Current evidence supports a hybrid “plasticity/fluid” scenario, where SCs dynamically assemble and disassemble according to cellular and metabolic conditions.

The precise role of SCs in OXPHOS remains unclear, but several hypotheses have been proposed (Figure 2). First, SCs assembly may support the assembly and stabilization of individual ETC complexes. In particular, evidence suggests the interaction of CIII and CIV with CI enhances the assembly and stability of CI [21,35,36,37]. However, CI dysfunction is rare in patients with CIII or CIV deficiencies, implying that only major disruptions in these complexes affect CI function [38], and some mature CI can be detected in the absence of SCs [39]. Second, as CI and CIII are primary sites for electron leak and, thus, superoxide production, the organization of these complexes into SCs might limit ROS output [40,41,42]. Specifically, SCs might enhance the efficiency of electron transfer by bringing ETC complexes closer together, reducing the diffusion distance for coenzyme Q and cytochrome c and facilitating direct substrate channeling, perhaps by isolating dedicated coenzyme Q and cytochrome c pools within the SCs [17,25,26,43], although this possibility has been widely debated [44,45,46,47,48,49]. Third, SCs formation may prevent protein aggregation within the IMM, where a high protein-to-lipid ratio could otherwise favor non-functional protein clustering, thereby ensuring functional organization within the IMM [22,44]. Finally, SCs might improve catalytic efficiency, with studies indicating that CI within the full CI + CIII_2_ + CIV respirasome is more active than it is in smaller assemblies [1,21].

Overall, efforts to determine the specific mechanistic effects of SCs assembly, particularly in mammalian cells, remain complicated by the lack of an agent that can prompt the disassembly of SCs without affecting the assembly and/or function of individual complexes. Interestingly, not all organisms rely on the formation of SCs to achieve efficient respiration. In *Drosophila melanogaster*, mitochondrial SCs are nearly absent, yet the mitochondria maintain high oxidative capacity. This efficiency is attributed to high concentrations of electron carriers and the presence of oxidation-resistant lipids, such as palmitoleic acid [50]. Further complicating the interpretation of experimental results, the specific metabolic conditions that trigger SCs assembly, stabilization, or disassembly, and even which SCs type is affected, are likely to differ by species, tissue, and cell type [22]. Defining the function of SCs in all contexts will require a thorough understanding of the molecular signals that regulate SCs assembly and maintenance.

## 4. Regulation of Mitochondrial Respiratory Supercomplex Assembly and Stability

Despite substantial progress in defining the structural organization of mitochondrial SCs, the temporal and mechanistic regulation of their assembly remains incompletely understood. It is still unclear whether SCs predominantly form through the association of fully assembled respiratory complexes or via sequential interactions of partially assembled intermediates and subunits, or whether both mechanisms operate depending on cellular context and metabolic state [2,3,4,5,20,21,51,52]. Moreover, the molecular pathways governing SCs assembly, composition, and stability—particularly in response to fluctuating bioenergetic demands—are only partially defined.

Multiple lines of evidence indicate that SCs organization is dynamically regulated by redox state, nutrient availability, and oxygen tension [22,53]. Early structural and biochemical studies identified redox-sensitive disulfide bonds within intermembrane space-exposed subunits of CI and CIII, suggesting that oxidative conditions may influence SCs assembly through conformational effects [28,54,55,56]. Subsequent work in human HEK293 and 143B osteosarcoma cells, as well as in mouse fibroblasts, demonstrated that mild oxidative stress can stabilize SCs, whereas severe oxidative stress destabilizes CI, CIII, and associated SCs, resulting in impaired mitochondrial respiration and increased ROS production [22,23,44,57,58]. Prolonged hypoxia or nutrient deprivation has also been shown to disrupt SCs integrity in U87 glioblastoma, U2OS osteosarcoma, C2C12 myoblasts, and mouse fibroblasts, although these effects are often reversible and strongly dependent on experimental conditions, duration of stress, and analytical methodology [6,7,34,59,60,61,62,63,64,65,66,67]. Collectively, these studies suggest that SCs organization responds dynamically to metabolic and redox cues, but that the functional outcomes vary markedly by cell type and context, complicating efforts to define universal regulatory principles.

Several proteins have been identified as key regulators of SCs assembly and maintenance. Among the most extensively characterized are supercomplex assembly factor 1 (SCAF1/COX7A2L), respiratory supercomplex factors Rcf1 and Rcf2 in yeast, and their mammalian orthologues HIGD1A and HIGD2A [22,53,57,58,68,69,70,71,72,73,74]. These factors regulate SCs through distinct mechanisms, often by influencing CIV biogenesis, activity, or its association with other respiratory complexes. SCAF1 stabilizes interactions between CIII_2_ and CIV and contributes to respirasome architecture, although it is not universally required for SCs formation and is absent in yeast and plants [21,27,57,58,69,71,73,74,75,76,77,78]. Experimental evidence from human HEK293, 143B, U87, and U2OS cells demonstrates that SCAF1 depletion disrupts SCs formation without consistently altering basal mitochondrial respiration, whereas SCAF1 upregulation under glucose deprivation enhances OXPHOS efficiency and supports cell survival [21,60,61,66,67,68]. These findings underscore the context- and cell type-dependent nature of SCAF1 function. In vivo studies further highlight this complexity: mouse COX7A2l variants with or without functional SCAF1 differentially affect SCs assembly, mitochondrial respiration, exercise capacity, fat deposition, and thermogenesis, while zebrafish models support a role for SCAF1 in whole-organism metabolic regulation [18,58,65,67,79].

Rcf1 and Rcf2 primarily regulate CIV assembly and activity in yeast, indirectly influencing SCs abundance under respiration-promoting growth conditions [69,71,73,75]. In mammalian systems, HIGD1A and HIGD2A contribute to CIV biogenesis and its incorporation into SCs, with evidence for differential roles in regulating CIV activity versus respirasome formation. Studies in human cancer cell lines and primary cells indicate that these proteins respond to hypoxia, nutrient stress, and redox cues, although their regulation is not uniform across models. For example, HIGD1A promoter hypermethylation has been reported in certain cancers, potentially limiting its inducibility, while HIGD2A shows context-dependent induction and function [53,80,81,82,83,84,85]. Negative regulation of SCs assembly is mediated by MCJ/DnaJC15, which suppresses CI activity and limits SCs formation in response to metabolic cues, thereby modulating mitochondrial membrane potential and ATP production [86].

Posttranslational modifications (PTMs) further fine-tune SCs dynamics. Phosphorylation of the CI subunit NDUFS4 via cAMP/PKA signaling enhances SCs formation and electron flux while limiting ROS production, as shown in mammalian cell systems [87]. Conversely, stress-induced cleavage of the mitochondrial fusion protein OPA1 disrupts inner mitochondrial membrane architecture and diminishes SCs integrity [88]. Modifications of cytochrome c have also been proposed to influence its interaction with CIII and CIV, potentially affecting electron transfer within SCs, although direct causal evidence remains limited [89].

Despite these advances, important controversies persist. Conflicting findings regarding SCAF1 function—where disruption of SCs does not always translate into impaired bioenergetics—raise fundamental questions about the causal relationship between SCs assembly and mitochondrial efficiency (Table 1). Similarly, it remains unclear whether observed SCs remodeling in cancer cells is a driver of metabolic adaptation or a secondary consequence of oncogenic signaling and altered mitochondrial dynamics. The functional significance of PTMs and regulatory proteins also appears highly dependent on cellular identity, metabolic state, and environmental stress, limiting generalization across systems. Moreover, how findings from transformed cell lines relate to primary patient-derived cells and in vivo physiology remains insufficiently explored.

Overall, current evidence indicates that SCs regulation integrates rapid signaling events with longer-term transcriptional, epigenetic, and metabolic programs. Resolving existing controversies will require standardized experimental frameworks, integration of diverse cell models—including primary and patient-derived systems—and systematic validation in animal models. Such efforts will be essential to establish causality between SCs organization and mitochondrial function and to clarify how SCs dynamics contribute to metabolic adaptation in physiology and disease.

Together, these findings suggest that mitochondrial SCs organization is not a static structural feature but a dynamically regulated node integrating metabolic stress, redox signaling, and environmental cues. From a translational perspective, this plasticity presents both opportunity and challenge: while cancer-associated remodeling of SCs may expose context-specific vulnerabilities, the strong dependence on cell type, metabolic state, and microenvironment complicates therapeutic targeting. Most current evidence derives from transformed cell lines or genetically engineered models, and direct validation in patient-derived systems and in vivo tumors remains limited. Moreover, the frequent disconnect between SCs disruption and bioenergetic failure raises questions about compensatory pathways and redundancy within the respiratory chain. Addressing these gaps will be essential to determine whether SCs represent actionable therapeutic targets or biomarkers of metabolic state, and to guide the rational development of strategies aimed at selectively exploiting SCs-dependent metabolic adaptations in cancer.

## 5. Mitochondrial SCs Stabilization as a Convergent Mechanism of Tumor Progression and Therapy Resistance in Cancer

While many unknowns remain regarding the species-, cell-, and stress-specific regulation of SCs and the effects thereof on mitochondrial bioenergetics, the stabilization of mitochondrial SCs is emerging as a unifying metabolic adaptation that supports tumor growth, tolerance to low oxygen and glucose concentrations, and resistance to therapy across multiple tumor types. Indeed, it has become apparent that malignant transformation and progression involve the heterogeneous reprogramming of the entire metabolic network in response to tumor-specific intracellular and environmental factors, including nutrient and O_2_ availability, as well as therapeutic toxicity. Although the downregulation of OXPHOS in favor of glycolysis allows cell survival in hypoxic conditions, many tumor cells maintain OXPHOS even while upregulating glycolysis, with transcription factors and other molecular regulators dynamically modulating the activity of each metabolic pathway to support survival, proliferation, and invasion as needed [91,92]. In particular, highly proliferative tumor cells, including cancer stem cells, rely on OXPHOS for energy. Furthermore, tumor cells often become dependent on OXPHOS when the intratumoral glucose supply has been depleted [93]. Increased OXPHOS efficiency may also limit the production of mitochondrial ROS production, attenuating ROS-induced cell damage and the therapeutic effects of radiation and chemotherapy [92]. As alluded to in the preceding section, emerging evidence indicates that the structural organization of SCs could enable cancer cells to sustain OXPHOS and anabolic metabolism despite the metabolic stress imposed by the tumor microenvironment or therapeutic interventions, revealing the potential relevance of targeting SCs assembly or function to selectively impair tumor metabolism, particularly in hypoxic or nutrient-deprived microenvironments.

## 6. Metabolic Effects of SCs-Regulating Factors in Cancer

Numerous publications have detailed the effects of SCs-regulating proteins, including HIGD1/2A, SCAF1, and MCJ/DnaJC15 on tumor cell survival, replication, aggression, and/or resistance to therapy in several types of cancer. Elevated expression of HIGD1A and HIGD2A mRNA has been detected in the tumors of patients with HCC, and high tumor expression of HIGD2A correlated with poor prognosis in patients [94,95]. In vitro and in vivo studies revealed that HIGD2A expression promotes OXPHOS and supports HCC progression and promotes tumor cell stemness [96]. Similarly, research has shown that HIGD1A expression, which is regulated by DNMT1-mediated hypermethylation, enhances tumor growth and metastasis. In contrast to HIGD2A, however, HIGD1A expression did not appear to promote OXPHOS [95]. Notably, knockdown of HIGD1A or HIGD2A did not affect normal liver cells in these studies.

In glioma cells, oncostatin M receptor (OSMR) localized to mitochondria and interacted with CI subunits NDUFS1 and NDUFS2 required for SCs assembly. Although the effects on SCs assembly were not specifically examined, loss of OSMR impaired mitochondrial respiration, elevated ROS, and sensitized the stem cells to radiation. Furthermore, knockdown of OSMR in the stem cells increased overall survival in xenograft models [97]. Pharmacologic inhibition of CI by mubritinib similarly enhanced radiotherapy efficacy selectively in patient-derived GBM stem cells by increasing ROS and DNA damage, extending survival in xenograft mouse models [98,99].

In addition, OPA1, which is recognized for controlling mitochondrial fusion, has been reported to promote SCs assembly and OXPHOS efficiency by regulating IMM morphology [100]. Upregulated expression of OPA1 in clinical samples is also associated with poor prognosis in all breast cancer subtypes and with relapse upon DNA-damaging chemotherapy in patients with TNBC [101,102]. Several groups have now reported that inhibition of OPA1 reduces growth and migration of metastatic TNBC cells and restores chemosensitivity after DNA-damaging chemotherapy, without affecting normal cells. However, the effects on SCs assembly were not directly examined [101,102,103].

Methylation of the MCJ gene encoding MCJ/DnaJC15 has been reported in ovarian cancer, as well as several other cancers. In ovarian cancer, higher levels of MCJ methylation correlated with lower overall survival and chemotherapeutic resistance. Conversely, overexpression of MCJ increased the sensitivity of ovarian cancer cells n (Sk-Ov-3) to chemotherapeutic agents [104,105,106]. In mice, global deletion of MCJ did not affect cellular function in physiologic conditions but led to increased SCs assembly in murine heart mitochondria [83].

**Figure 3:**
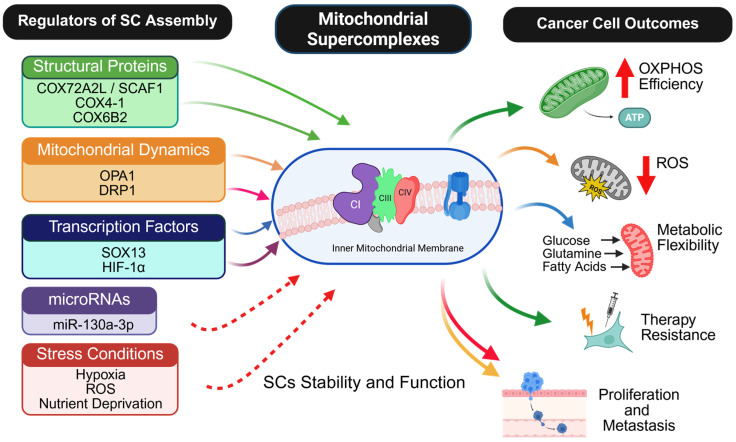
Major regulators and cancer cell outcomes of mitochondrial SCs. Structural proteins (e.g., COX7A2L/SCAF1, COX4-1, COX6B2), mitochondrial dynamics regulators (OPA1, DRP1), transcriptional programs (e.g., HIF-1α, SOX13), microRNAs, and tumor-associated stress conditions converge to regulate the assembly, stability, and function of mitochondrial SCs within the inner mitochondrial membrane. Stabilized SCs support OXPHOS efficiency, limit mitochondrial ROS production, enhance metabolic flexibility, and promote tumor cell proliferation, therapy resistance, and metastasis. Green arrows indicate stabilizing effects on SCs assembly or downstream cancer phenotypes. Orange arrows indicate modulatory regulation. Blue arrows denote transcriptional control. Purple arrows indicate post-transcriptional regulation. Red arrows represent stress-induced or destabilizing influences. Solid arrows indicate experimentally validated, direct regulatory relationships. Dashed arrows indicate indirect, inferred, or context-dependent effects.

Whereas the studies discussed above implicate SCs-regulating proteins in metabolic adaptations associated with tumor progression, survival, and therapy resistance, many do not directly assess mitochondrial SCs architecture or composition, highlighting an important gap between functional observations and structural characterization. Nonetheless, accumulating evidence supports the view that mitochondrial SCs are dynamic assemblies whose organization is influenced by multiple regulatory layers, including structural subunits and assembly factors, mitochondrial dynamics, transcriptional and post-transcriptional mechanisms, and microenvironmental conditions such as hypoxia, oxidative stress, and nutrient availability. To integrate these diverse regulatory inputs and the cancer-associated phenotypes with which SCs organization has been linked, Figure 3 provides an overview of the major regulators of mitochondrial SCs in cancer and summarizes reported associations between SCs stabilization and cancer cell outcomes, including altered oxidative phosphorylation capacity, redox balance, metabolic flexibility, stress tolerance, and therapy resistance. This conceptual overview sets the stage for the following section, which examines how SCs-related metabolic features have been described across different cancer types and experimental systems.

## 7. Regulation of SC Organization in Cancer

Although the molecular principles governing mitochondrial SCs assembly are broadly conserved, emerging evidence indicates that cancer cells exploit SCs regulation in tumor-specific ways that reflect lineage, metabolic demand, and microenvironmental stress. Across tumor types, most studies rely on established cancer cell lines, with more limited but growing validation in patient-derived models and clinical specimens. Importantly, only a subset of studies directly assesses SCs assembly or architecture using BN-PAGE, cryo-EM, or complex-specific immunoprecipitation; many infer SCs involvement indirectly from changes in OXPHOS activity, ROS production, or mitochondrial morphology. Below, we highlight tumor-specific features of SCs regulation while explicitly distinguishing direct evidence from inferred associations.

### 7.1. Pancreatic Ductal Adenocarcinoma

Pancreatic ductal adenocarcinoma (PDAC) develops within a profoundly hypoxic and nutrient-deprived microenvironment, yet tumor cells retain robust proliferative capacity and high metabolic plasticity. Despite long-standing assumptions that PDAC relies predominantly on glycolysis, accumulating evidence indicates that PDAC cells maintain active OXPHOS, particularly under metabolic stress. In human PDAC cell lines and patient-derived tumor samples, Masoud et al. demonstrated that PDAC cells dynamically shift between glycolytic and OXPHOS states in response to environmental cues. Notably, intratumoral OXPHOS activity varied substantially across patients and inversely correlated with overall survival. Pharmacologic inhibition of CI using phenformin reduced OXPHOS in high-respiration PDAC cells and selectively sensitized these tumors to gemcitabine therapy in xenograft mouse models, establishing a functional link between mitochondrial respiration and therapeutic response [107].

More direct evidence implicating mitochondrial SCs in PDAC metabolic adaptation was provided by Hollinshead et al. Using human PDAC cell lines cultured under severe hypoxia and nutrient limitation, the authors demonstrated that preservation of mitochondrial morphology and OXPHOS capacity depended on SCAF1-mediated stabilization of CIV-containing SCs, including respirasomes. Genetic deletion of COX7A2L (SCAF1) disrupted SCs assembly, reduced mitochondrial efficiency, increased ROS-mediated damage, and selectively impaired hypoxic tumor growth both in vitro and in orthotopic mouse models [70]. These findings establish SCs stabilization as a causal mechanism enabling PDAC cells to sustain OXPHOS under extreme metabolic stress.

Complementary work by Zhang et al. revealed additional context-dependent roles for SCs in PDAC. In human PDAC cell lines under normoxic but glutamine-limited conditions, SCAF1-driven SCs assembly reduced dependence on glutamine oxidation by favoring electron flux from CI to CIII, thereby limiting reliance on CII-supported respiration. Although this metabolic configuration constrained proliferation, it enhanced tumor cell survival in glutamine-poor microenvironments characteristic of PDAC tumors. Further suggesting the clinical relevance of SCAF1 in PDAC, their bioinformatics analysis of data in The Cancer Genome Atlas confirmed that COX7A2L mRNA is upregulated in human PDAC tumors [49]. These findings collectively identify SCs as essential for tumor cell adaptation in hypoxic or nutrient-deprived environments and thus the regulation of metabolic plasticity and tumor progression and survival in PDAC. Importantly, the genetic alterations common in PDAC do not appear to influence OXPHOS status [107], suggesting that SCs-targeted treatments may be broadly applicable for patients with PDAC.

### 7.2. Breast Cancer

Mitochondrial OXPHOS has emerged as a critical determinant of tumor aggressiveness and therapy resistance in multiple breast cancer subtypes, including triple-negative breast cancer (TNBC) and HER2high tumors. In both contexts, mitochondrial SCs contribute to the maintenance of mitochondrial efficiency, particularly under hypoxic conditions. Studies using HER2high breast cancer cell lines demonstrated that SCs organization supports sustained OXPHOS during hypoxia, preserving biosynthetic capacity required for rapid proliferation [108,109].

Clinical relevance of SCs regulation in breast cancer is underscored by analyses of patient samples showing that COX7A2L (SCAF1) mRNA is upregulated across breast cancers of varying estrogen receptor and HER2 status, with elevated expression correlating with poor patient survival, including after tamoxifen treatment [110]. Mechanistic studies in hormone-responsive MCF-7 cells revealed that 17β-estradiol induces SCAF1 expression, promoting SCs assembly, enhancing OXPHOS, reducing mitochondrial ROS production, and increasing glutathione synthesis under both normoxic and hypoxic conditions. These metabolic adaptations supported enhanced tumor growth in vitro and in vivo and conferred hormone-independent growth in mouse xenograft models, implicating SCs assembly in endocrine therapy resistance [110].

Conversely, disruption of SCs through genetic depletion or siRNA-mediated inhibition of COX7A2L, or through pharmacologic targeting of mitochondrial CI, suppressed tumor growth in MCF-7 and TNBC (MDA-MB-231) cells both in vitro and in vivo [110,111,112]. Notably, treatment with MitoTam, a mitochondria-targeted tamoxifen derivative, impaired SCs-supported OXPHOS and tumor progression without inducing systemic toxicity in mouse models, highlighting the translational potential of targeting mitochondrial organization rather than individual ETC complexes [112]. Similarly, inhibition of mitochondrial iron metabolism using mitochondria-targeted deferoxamine reduced SCs assembly, increased mitochondrial ROS, and suppressed tumor growth and metastasis in both hormone-dependent and TNBC models [91].

The relationship between SCs and tumor progression in breast cancer is further complicated by inflammatory signaling within the tumor microenvironment. In MCF-7 and MDA-MB-231 cells, TNF-α disrupted SCs abundance and activity, with a more pronounced effect in TNBC cells, leading to reduced OXPHOS and increased mitochondrial ROS. Paradoxically, TNF-α enhanced tumorigenicity in TNBC while suppressing growth in MCF-7 cells. Analysis of Tumor Immune Estimation Resource (TIMER) datasets revealed that high TNF-α expression in human basal breast cancer correlates with reduced expression of key CI and CIII subunits and poorer survival outcomes. Restoration of OXPHOS using hemin mitigated TNF-α-driven tumorigenic effects, suggesting that inflammatory suppression of ETC biogenesis indirectly destabilizes SCs and reshapes metabolic phenotypes in a subtype-specific manner [92].

Finally, mitochondrial ultrastructure emerges as an additional determinant of SCs function in breast cancer. OPA1, a regulator of mitochondrial fusion and cristae organization, promotes SCs assembly and OXPHOS efficiency by maintaining IMM architecture. Elevated OPA1 expression correlates with poor prognosis across breast cancer subtypes and with relapse following DNA-damaging chemotherapy in TNBC patients [113,114,115]. Inhibition of OPA1 reduced growth, migration, and chemoresistance in metastatic TNBC models without affecting normal cells, although direct effects on SCs assembly were not quantified [113,115,116].

Together, these findings establish mitochondrial SCs as central structural and functional mediators of metabolic flexibility, hypoxia tolerance, and therapy resistance in breast cancer. SCs stabilization enables tumor cells to sustain OXPHOS under adverse conditions, reinforcing SCs as a convergent and therapeutically actionable metabolic adaptation.

### 7.3. Hepatocellular Carcinoma

Hepatocellular carcinoma (HCC) is among the most hypoxic solid tumors [93], creating strong selective pressure for mitochondrial adaptations that sustain bioenergetic and redox homeostasis. Consistent with this environment, elevated expression of HIGD1A and HIGD2A mRNA has been detected in tumor samples from patients with HCC, with high tumor expression of HIGD2A correlating with poor prognosis [117,118].

Functional studies using human HCC cell lines, including HepG2, Huh7, and MHCC97H, demonstrated that HIGD2A knockdown impaired mitochondrial OXPHOS and suppressed tumor cell proliferation and survival in vitro, while also reducing tumor growth in vivo in mouse xenograft models, directly linking enhanced mitochondrial respiration to HCC tumorigenicity [117]. Additional in vitro assays further indicated that HIGD2A promotes tumor cell stemness. Although these effects were hypothesized to arise from increased mitochondrial SCs assembly driven by HIGD2A-mediated CIV biogenesis, the relative abundance or organization of SCs was not directly examined in these studies [117].

In parallel, analyses of human HCC tumor tissues and derived cell lines revealed that upregulation of HIGD1A also enhances tumor growth and metastasis, with expression regulated by DNMT1-mediated promoter hypermethylation. In contrast to HIGD2A, however, HIGD1A expression did not appear to promote OXPHOS, underscoring mechanistic divergence among SCs-regulating factors in HCC [118]. Notably, knockdown of neither HIGD1A nor HIGD2A adversely affected normal hepatocytes, suggesting tumor-selective metabolic dependencies.

The expression of COX7A2L (SCAF1) mRNA is likewise upregulated in HCC patient tumors and correlates with poor prognosis and increased metastatic potential. In vitro and in vivo functional studies using human hepatoma cell lines and mouse xenograft models, conducted under normoxic conditions, demonstrated that SCAF1 promotes tumor growth and metastasis by inducing mitochondrial ROS production and activating NF-κB signaling, thereby enhancing cell cycle progression and epithelial-to-mesenchymal transition while suppressing apoptosis. Both SCAF1 expression and its tumor-promoting effects were inhibited by a miR-130a-3p mimic. In patient samples, miR-130a-3p expression was downregulated in HCC tumors and correlated positively with overall survival and inversely with SCAF1 expression [94,95,96].

Supporting a broader regulatory role for SCAF1-linked pathways, miR-130a-3p is also downregulated in breast cancer tumors, where in vitro studies using breast cancer stem-like cells suggested tumor-suppressive effects. Reduced miR-130a-3p expression was further observed in tumors from patients with advanced, chemotherapy-resistant breast cancer, and overexpression of this miRNA restored doxorubicin sensitivity in MCF-7/Adr cells [97]. However, context-dependent effects complicate translational interpretation: miR-130a-3p upregulation was reported in cisplatin-treated HCC patient tumors and promoted cisplatin resistance in Huh7 cells [99], with similar resistance phenotypes observed in esophageal squamous cell carcinoma cell lines [98,100]. Additionally, miR-130a-3p has been identified as a marker of high-grade cervical cancer and shown to promote disease progression in clinical samples and cell-based models [101,102,103].

Collectively, studies using HCC cell lines, patient tumor specimens, and mouse xenograft models implicate HIGD2A-, HIGD1A-, and SCAF1-associated pathways in shaping mitochondrial metabolism, redox signaling, and tumor progression in HCC. While several findings are consistent with altered SCs assembly or function, direct biochemical interrogation of SCs organization remains limited, highlighting an important gap that must be addressed to define the precise contribution of mitochondrial SCs to HCC pathogenesis and to evaluate their therapeutic tractability across cancer types.

### 7.4. Endometrial Cancer

Analyses of patient-derived tumor tissues have revealed that COX7A2L (SCAF1) expression is upregulated in endometrial cancer, implicating mitochondrial SCs regulation in disease progression [110]. Functional studies using human endometrial cancer Ishikawa cells demonstrated that SCAF1 acts as an SCs assembly-promoting factor, stabilizing CI-containing SCs even under hypoxic conditions. Biochemical and functional analyses in these cells showed that SCAF1-dependent SCs stabilization was associated with enhanced mitochondrial OXPHOS efficiency, reduced mitochondrial ROS production, and improved cell growth and survival during hypoxia in vitro. The physiological relevance of these findings was further supported by in vivo tumor growth in mouse xenograft models, in which SCAF1 expression promoted endometrial tumor growth under hypoxic stress [110].

Together, these studies using clinical specimens, human endometrial cancer cell lines, and mouse models provide direct evidence that SCAF1-mediated stabilization of CI-containing SCs supports mitochondrial bioenergetics, redox control, and hypoxia tolerance in endometrial cancer. Importantly, this work represents one of the clearest demonstrations across tumor types in which SCs assembly was directly assessed, strengthening the causal link between SCs stabilization and tumor metabolic fitness.

### 7.5. Lung Cancer

Lung cancers, including non-small cell lung cancer (NSCLC), exhibit substantial metabolic heterogeneity, with subsets of tumors maintaining high mitochondrial OXPHOS activity despite hypoxic or nutrient-limited microenvironments. Studies using human NSCLC cell lines (A549, H1299), lung adenocarcinoma cell lines (H1975, HCC827), and xenograft mouse models have shown that OXPHOS dependency correlates with aggressive behavior and therapy resistance, particularly in tumors with elevated mitochondrial mass and respiratory capacity [104,105].

While many investigations infer mitochondrial adaptations based on respiration, ROS production, or cristae morphology, a growing body of evidence implicates mitochondrial SCs as structural determinants of this metabolic phenotype.

In human lung adenocarcinoma cell lines, overexpression of COX6B2—but not its somatic isoform COX6B1—enhanced CIV-containing SCs assembly, increased OXPHOS without elevating mitochondrial ROS production, and conferred a proliferative advantage, particularly under hypoxic conditions. Conversely, genetic depletion of COX6B2 attenuated OXPHOS, collapsed mitochondrial membrane potential, and induced cell death or senescence in vitro, while significantly suppressing tumor growth in mouse xenograft models [105]. In parallel, treatment of doxorubicin- or cisplatin-resistant metastatic lung cancer cell lines with MitoTam disrupted SCs and restored sensitivity to mitochondrial ROS-inducing chemotherapeutics in vitro [104].

Collectively, these studies across lung cancer cell lines and in vivo tumor models provide direct biochemical and functional evidence that SCs stabilization supports OXPHOS maintenance, redox homeostasis, and therapy resistance in lung adenocarcinoma.

### 7.6. Glioblastoma

Glioblastoma exhibits pronounced metabolic heterogeneity yet retains a strong dependence on OXPHOS, particularly within hypoxic and nutrient-limited tumor niches. Glioblastoma stem-like cells (GSCs), which contribute to tumor recurrence and therapeutic resistance, rely on OXPHOS supported by mitochondrial SCs to maintain survival under metabolic stress. In patient-derived GSCs, the oncostatin M receptor (OSMR) was shown to localize to mitochondria and interact with CI subunits NDUFS1 and NDUFS2, which are required for SC integrity. Genetic knockdown of OSMR impaired mitochondrial respiration, increased mitochondrial ROS, and sensitized GSCs to ionizing radiation, resulting in prolonged survival in orthotopic xenograft mouse models [119]. Although SCs assembly was not directly quantified in this study, the disruption of CI function and respiratory efficiency is consistent with compromised SCs-supported electron transport.

Pharmacologic targeting of CI further supports a functional role for SCs-dependent OXPHOS in glioma. In patient-derived GSCs, inhibition of CI with mubritinib increased ROS accumulation and DNA damage, selectively enhancing radiosensitivity and extending survival in xenograft mouse models without comparable toxicity to nonmalignant cells [120,121]. These findings parallel observations in other tumor types, indicating that sustained OXPHOS—likely facilitated by SCs organization—contributes to therapy resistance.

More direct mechanistic evidence linking SCs assembly to glioma metabolism comes from studies of cytochrome c oxidase subunit 4 isoform 1 (COX4-1). Using patient-derived and established glioblastoma cell lines, shRNA-mediated depletion of COX4-1 caused a marked reduction in the assembly of CIV-containing SCs, accompanied by impaired CIV activity, reduced respiratory capacity, and increased oxidative stress. Conversely, COX4-1 overexpression promoted SCs assembly, enhanced OXPHOS efficiency, and supported mitochondrial function, particularly under hypoxic conditions [122,123]. These effects translated in vivo, as COX4-1 knockdown suppressed tumor growth in glioma mouse models, while elevated COX4-1 expression supported tumor progression [124].

Together, these studies establish a causal link between CIV composition, SCs assembly, and metabolic fitness in glioblastoma. Glioma cells exploit SCs stabilization to sustain OXPHOS, limit ROS-mediated damage, and resist therapeutic stress. Collectively, these findings position mitochondrial SCs—rather than individual ETC complexes alone—as structurally integrated and functionally relevant targets for disrupting metabolic plasticity and therapy resistance in glioblastoma.

### 7.7. Gastric Cancer

Gastric cancer cells must adapt to fluctuating oxygen levels and high oxidative stress within the tumor microenvironment, conditions that challenge mitochondrial bioenergetics. A recent study by Yang et al. used ferroptosis resistant human gastric cancer cell lines SNU 668 and SNU 484 to identify transcriptional regulation of SCAF1 by the transcription factor SOX13 as a key regulator of mitochondrial SCs assembly and cell survival [125]. SOX13 was upregulated relative to ferroptosis sensitive cells and promoted the formation of SCs by transcriptionally upregulating SCAF1, thereby enhancing mitochondrial OXPHOS and cell resistance to oxidative damage.

Functional perturbation using SOX13 genetic silencing (shRNA/siRNA) in SNU 668 and SNU 484 cells reduced SCAF1 expression, impaired SCs assembly, decreased OXPHOS capacity, and sensitized the cells to ferroptosis mediated therapies.

Complementing these in vitro findings, analysis of gastric cancer patient cohorts who received cisplatin-based adjuvant chemotherapy demonstrated that high tumor expression levels of both SOX13 and SCAF1 correlated inversely with overall survival, indicating translational relevance of this regulatory axis. Collectively, data from gastric cancer cell models and clinical specimens’ position SOX13–SCAF1-mediated SCs assembly as a central mitochondrial adaptation that supports redox resilience and therapy resistance, highlighting a promising metabolic vulnerability for therapeutic exploitation [125].

### 7.8. Ovarian Cancer

Ovarian cancer progression and chemoresistance are closely linked to mitochondrial metabolic adaptations. Studies in human ovarian cancer cell lines, including SKOV3 and related platinum-resistant derivatives, demonstrate that enhanced OXPHOS activity correlates with poor clinical outcome and resistance to chemotherapy [126,127,128,129]. MCJ/DnaJC15 has been shown to regulate mitochondrial respiration and oxidative stress in mammalian cells [86], and epigenetic silencing of the MCJ gene has been reported in ovarian cancer as well as in melanoma, pediatric brain tumors, and Wilms’ tumors.

In ovarian cancer, higher levels of MCJ methylation correlated inversely with overall survival and with chemotherapeutic response in patients. Conversely, overexpression of MCJ/DnaJC15 increased chemosensitivity in ovarian cancer cell lines in vitro, linking MCJ-mediated suppression of mitochondrial respiration to therapeutic response [126,127,129,130]. In MCJ knockout mice, loss of MCJ did not affect normal tissue function under physiologic conditions but led to increased SCs assembly in cardiac mitochondria, illustrating tissue-specific consequences of SCs regulation [86].

Although SCs assembly was not directly quantified in most ovarian cancer models, the convergence of cell line-based functional studies, patient correlative data, and mouse genetic models strongly supports a role for SCs stabilization in ovarian tumor metabolism and chemoresistance.

### 7.9. Leukemia

Relative to normal hematopoietic cells, acute myeloid leukemia (AML) cell lines exhibit elevated accumulation of SCs, supporting efficient OXPHOS. Increased SCs abundance was also detected in cell samples from some patients with AML, and this increase correlated with enhanced expression of the mitochondrial peptidase neuroly-sin (NLN). In NB4 and OCI-AML2 cells, expression of NLN and its partner leucine zipper EF-hand containing transmembrane protein 1 (LETM1) was essential for SCs assembly. Of therapeutic implication, pharmacologic inhibition of NLN selectively impaired AML metabolism and the survival of AML cells and stem cells in vitro and in vivo, without inducing toxicity in normal hematopoietic stem cells [36].

Collectively, these studies underscore mitochondrial SCs as central metabolic hubs whose targeted disruption offers a promising strategy to selectively impair malignant cells while sparing normal tissues, paving the way for more effective and precise cancer treatments.

## 8. Mitochondrial SCs as a Unifying Metabolic Framework and Therapeutic Leverage Point in Cancer

The collective evidence across tumor types positions mitochondrial SCs as a convergent metabolic adaptation that enables cancer cells to sustain OXPHOS, limit oxidative damage, and maintain anabolic capacity under hypoxia, nutrient limitation, and therapeutic stress. Genetic perturbations, pharmacologic interventions, and in vivo models increasingly support a causal role for SCs organization in tumor metabolic fitness and therapy resistance, rather than a purely correlative association. SCs stabilization is thus a point of therapeutic leverage, distinct from broad inhibition of the electron transport chain, as it can selectively impair tumor bioenergetics while sparing normal tissues.

Preclinical studies targeting SCs support this potential. While ETC inhibitors such as rotenone, antimycin A, metformin, IACS-010759, and devimistat have demonstrated anti-tumor activity, clinical translation has been limited by off-target toxicity or suboptimal responses [131,132,133,134].

To date, only one agent shown to affect SCs assembly has been assessed in a clinical study in patients with cancer. In preclinical studies, this agent, the mitochondrially targeted derivative of tamoxifen known as MitoTam, selectively disrupted SCs formation in breast cancer cells overexpressing HER2 [112] and enhanced the efficacy of radiotherapy in radioresistant head and neck cancer cell lines by overcoming the high antioxidant potential of these cells [135]. In a phase I/Ib trial involving patients with metastatic solid tumors, MitoTam was generally well-tolerated and induced a clinical response in 37% of patients. Interestingly, these responses were primarily observed in patients with metastatic renal cancer. A follow-up study of MitoTam in renal cancer cell lines confirmed that this agent inhibits CI-dependent mitochondrial respiration and induces necroptosis. In a syngeneic model of renal cancer cell-derived tumors, MitoTam exhibited selective toxicity for tumor cells, increased overall survival, and prevented lung metastasis. These results support the potential of MitoTam as a therapeutic agent targeting SCs [105,128,133,136]. However, agents that can target the assembly of SCs, specifically, without inhibiting the individual complexes have not yet been identified.

## 9. Conclusions

Mitochondrial SCs are dynamic, multifactorial assemblies essential for maintaining mitochondrial efficiency, redox balance, and structural integrity. Their regulation involves a coordinated interplay of structural proteins, lipids, and signaling pathways that allow tumor cells to adapt to metabolic stress and protect against bioenergetic failure. This adaptation underpins more aggressive and therapy-resistant phenotypes across diverse cancer types.

Although SCs stabilization is consistently observed across multiple tumors and largely promotes cancer cell survival and resistance to therapy, the precise mechanisms driving SCs remodeling—including transcriptional programs, posttranslational modifications, and signaling pathways—remain incompletely defined. Likewise, how tumor-specific metabolic stressors, such as hypoxia, acidosis, or nutrient deprivation, selectively induce SCs reorganization is not fully understood. The heterogeneity of SCs composition across tumor microenvironments and disease stages is also under characterized, leaving open questions about SCs evolution during metastasis or in response to therapy.

From a therapeutic perspective, pharmacologic targeting of SCs has begun to show promise. For example, the CI-targeting agent MitoTam can destabilize SCs and has exhibited clinical efficacy in metastatic renal cancer, but broader activity across tumor types has been limited. Future efforts will require the development of predictive biomarkers to identify SCs-dependent tumors and strategies to selectively target SCs assembly without disrupting individual electron transport chain complexes.

Collectively, these findings position mitochondrial SCs as both a central regulator of tumor metabolic fitness and a tractable therapeutic vulnerability, offering a path toward precision strategies that selectively disrupt cancer cell bioenergetics while sparing normal tissues.

## Figures and Tables

**Table 1:** Impact of SCs assembly factors on mitochondrial structure and function.

Study	Model	Tissue- Cell Type	Effect on SCs	Effect on Mitochondrial Bioenergetics and Phenotype	Genetic Background
Perez-Perez et al. [63]	SCAF1 KD	143B cells	Loss of CII2+CIV SCs	No change in OCR	
Lobo-Jarne et al. [34]	COX7A2L-KO	HEK293T and U87MG cells	Loss of CII2+CIV SCs and some large SCs (CI+CIII2+CIV2-4). WT SC phenotype restored by ectopic expression of long but not short COX7A2L	No change in bioenergetics under physiologic conditions or in nutritional stress (galactose) or with heat shock or oxidative stress	
Balsa et al. [68]	COX7A2L-KO	U2OS cells	Decrease in abundance of CI+CIII2+CIVn respirasomes vs. WT cells	No change in OCR in cells cultured in glucose. Decrease in bioenergetics in cells cultured in galactose	
Zhang et al. [49]	COX7A2L-KO	HEK293T, C2C12 and 3T3-L1 cells	Loss of CIII2+CIV SCs and large SCs (CI+CIII2+CIV2-4)	Did not affect ATP production or glucose metabolism. Enhanced CII-mediated respiration	
Fernandez-Vizarra [61]	COX7A2L-KO	HEK293T cells	30–40% decrease in respirasomes CI+CIII2+CIV. Loss of CIII2+CIV SCs		
	COX7A2-KO	HEK293T	Presence of CI+CIII2+CIV respirasome bound only to SCAF1. Increased abundance of CIII2+CIV SCs		
	WT	HEK293T	60–70% of CI+CIII2+CIV respirasomes contain COX7A2, 30–40% contain SCAF1		
		HEK293T	Loss of CIII2+CIV SCs. No change in the abundance of CI+CIII2+CIV respirasomes containing COX7A2	No change in mitochondrial bioenergetics, even when cultured with galactose instead of glucose	
Lapuente et al. 2013 [18]	SCAF1 short and SCAF1 long	Fibroblasts	SCAF1 short: No CIV-containing SCs SCAF1 long: CIV-containing SCs		SCAF1 short: C57BL/6J and BALB/c mice SCAF1 long: 129sv mice
Benegiamo 2022 [79]	Cox7a2l DD allele	Skeletal muscle and Liver	Increase in CIII2+CIV SCs with exercise in muscle. No change with exercise in liver	Muscle: Increased mitochondrial bioenergetics and increased lean mass Animals had lower body weight and higher food intake. Liver: Not reported	C57BL/6J
	COX7A2L variant with 10-bp insertion	Myotubes	Increased stability of COX7A2L mRNA; increased SCAF1 expression. Increased abundance of SCs under galactose.	Enhanced bioenergetics under galactose. Subjects had lower body fat and improved cardiorespiratory fitness	
Williams et al. 2016 [67]	B6 allele	Heart	Absence of III2+IV1, I+III2+IV2, and I+III2+IV3 SCs		B6 background vs. D2 background
		Liver	Reduced abundance of III2+IV1, I+III2+IV2, and I+III2+IV3 SCs		
Ikeda et al. 2013 [62]	Cox7rpKO mice vs. WT	Fibroblasts		Decreased bioenergetics	C57Bl/6
		Muscle	Decreased SC formation	Decreased muscle strength and heat production	
	COX7RP-TG mice vs. WT	Muscle	Increased SC formation	Increased muscle strength and heat production	
Shiba et al. 2017 [65]	Cox7rpKO mice	Liver	Increased SC formation	Increased ATP production	
Garcia-Poyatos 2020 [58]	scaf1-/- zebrafish		Loss of CIII2+CIV SCs	Decreased bioenergetic efficiency. Small size, abnormal fat distribution, and female infertility	
Benegiamo et al. 2022 [79]	COX7A2L variant with 10-bp insertion	Myotubes	Increased stability of COX7A2L mRNA; increased SCAF1 expression and abundance of SCs	Enhanced bioenergetics. Lower body fat and improved cardiorespiratory fitness	Human
	Cox7a2l DD allele	Muscle	Increased abundance of SCs in skeletal muscle after 5 weeks of exercise training	Increased max O2 consumption, muscle mass, and increased energy expenditure during activity	C57BL/6
Ren et al. 2024 [90]	COX7A2L-KD	H9C2 cells		Increased hypoxia-induced mitochondrial ROS production	

## Data Availability

No new data were created or analyzed in this study.
